# Orthopaedic corticosteroid injections and risk of acute coronary syndrome: a cohort study

**DOI:** 10.3399/bjgp20X713945

**Published:** 2021-01-12

**Authors:** Katharine Thomas, Yochai Schonmann

**Affiliations:** Department of Family Medicine, Tel Aviv University, Tel Aviv, Israel.; Healthcare Quality Indicators Program for Clalit Health Services Hospitals, Department of Quality Measurements and Research, Clalit Health Services, Tel Aviv; Sial Research Center, Division of Community Health, Ben-Gurion University of the Negev, Beer-Sheva, Israel.

**Keywords:** acute coronary syndrome, corticosteroid injections, intra-articular injections, orthopaedics, primary health care, rheumatology

## Abstract

**Background:**

Corticosteroid injections (CSIs) are a common treatment for arthritis and other musculoskeletal conditions.

**Aim:**

To determine whether there is an increased incidence of acute coronary syndrome (ACS) following intra-articular and soft-tissue CSI.

**Design and setting:**

Cohort study in an urban primary care orthopaedic clinic.

**Method:**

Data were reviewed from all patients aged ≥50 years and seen by orthopaedic specialists between April 2012 and December 2015, including CSI, hospital admission in the week following the orthopaedic visit, and cardiovascular risk factors. The incidence of an ACS-associated hospital admission was compared between visits in which patients received CSIs and visits in which patients did not.

**Results:**

A total of 60 856 orthopaedic visits were reviewed (22 131 individual patients). The mean age was 70.9 years (standard deviation [SD] = 10.8), and 66.5% were female. Injections were administered in 3068 visits (5.1%). In the week following the visit there were 25 ACS hospital admissions (41 per 100 000 visits); seven events were after visits with an injection, and 18 were after non-injection visits. Patients who had received an injection were more likely to experience a subsequent ACS. (227 versus 31 events per 100 000 visits, odds ratio [OR] = 7.3; 95% confidence interval [CI] = 2.8 to 19.1). The association between receiving a CSI and ACS remained similar when the analysis was restricted to subgroups defined by age, sex, and cardiovascular risk factors.

**Conclusion:**

CSI for musculoskeletal conditions may substantially increase the risk of ACS in the week following the injection. Although the absolute risk of ACS is small, the effect size appears to be clinically significant.

## INTRODUCTION

Intra-articular and soft-tissue corticosteroid injection (CSI) is a common treatment for arthritis and other inflammatory musculoskeletal conditions.^1^^–^^3^ In the US alone, several million injections are administered annually.^4^ CSIs are generally considered safe and effective, particularly in providing short-term symptom relief.^5^^,^^6^ Complications such as local trauma and infection have been reported, but their incidence is low.^7^^–^^9^

The existence of serious endocrinological side effects from glucocorticoid steroid treatments was acknowledged soon after their introduction as a new and effective anti-inflammatory medication.^10^ Forty years later, Maxwell *et al*^11^ raised the possibility that systemic corticosteroids also increase coronary heart disease. Corticosteroid treatment has been previously associated with risk factors for cardiovascular disease such as hypertension, hyperlipidaemia, and hyperglycaemia.^12^^,^^13^ Oral corticosteroid treatment may also be an independent risk factor for ischaemic events, particularly during treatment.^14^^,^^15^ To the best of the authors’ knowledge, however, the role of intra-articular and soft-tissue CSI as a potential risk factor for cardiovascular events has not been previously explored. Although hyperglycaemia and elevated blood pressure have been reported to occur in the hours after articular injections,^16^^,^^17^ several systemic reviews that considered the side effects of intra-articular and soft- tissue CSI did not mention ischaemic heart disease.^6^^,^^7^^,^^18^^,^^19^ Furthermore, guidelines for the treatment of arthritis and inflammatory musculoskeletal conditions recommending the use of CSI published by Osteoarthritis Research Society International, the American College of Rheumatology, and the National Institute for Health and Care Excellence do not mention cardiac ischaemia as a potential side effect.^1^^,^^2^^,^^20^

Cardiovascular disease continues to be a significant cause of morbidity and mortality worldwide;^21^ in Israel, 25 000 patients are admitted to hospital each year with acute coronary syndrome (ACS).^22^ Elucidating a possible link between a common orthopaedic procedure such as CSI and ischaemic events may be relevant to patients, clinicians, and policymakers. Therefore, this study aimed at examining whether intra-articular and soft-tissue CSI injections are associated with an increased incidence of acute coronary events during the week following their administration.

## METHOD

### Setting and data sources

Israel has a national health insurance scheme that guarantees medical care to all citizens through four healthcare providers, of which Clalit Health Services (CHS) is the largest. CHS members have comparable health characteristics with those of other healthcare providers,^23^^,^^24^ and the CHS population has been used as a proxy for the national population in various published reports.^25^^–^^28^ CHS has operated a centralised computerised personal medical file since the late 1990s, linking each patient’s individual national identity number with data from multiple sources, including primary care physicians, specialty clinics in the community, hospital records, laboratories, and pharmacies.^29^

**Table:** How this fits in

Intra-articular and soft-tissue corticosteroid injection (CSI) is a common treatment for orthopaedic conditions, often performed in primary care. CSIs are believed to have a low incidence of minor local side effects, and no known association with acute ischaemic cardiac events. This study suggests an increased incidence of acute coronary syndrome (ACS) in the week following CSI. The possible association of a common treatment and a life-threatening side effect is of importance to patients, clinicians, and policymakers.

This study was set in the orthopaedic clinic of the main CHS specialist consultation centre in Tel Aviv. The orthopaedic clinic is open 46 hours a week, and permanently staffed by eight orthopaedic specialists and a rotating staff of orthopaedic specialists from other clinics and hospitals who work on an *ad hoc* basis. Community- based orthopaedic consultations are a primary care service in Israel for which the majority of patients will have self-referred; a minority will have been referred by a different specialist or by their family physician. The orthopaedic consultation requires a small co-payment, and waiting lists are relatively short (a few days to several weeks). Orthopaedic specialists are reimbursed separately for office-based procedures like CSI (fee-for-service), based on the procedure codes that they record in the medical file. This file also allows the orthopaedic specialist access to the patients’ medical record including past medical history and medication use.

All patients aged ≥50 years who consulted with an orthopaedic specialist between 1 April 2012 and 31 December 2015 were included. The CHS chronic disease register was used to extract clinical information for the following conditions: smoking, ischaemic heart disease, hypertension, diabetes, hyperlipidaemia, obesity, and heart failure (see Supplementary Box S1 for details).^29^ CSI was considered to have been carried out during the orthopaedic consultation when the associated injection procedure code appeared in the medical file. During the study period the only non-corticosteroid product available for orthopaedic injections was the hyaluronic acid Arthrease^®^ Patients who had filled an Arthrease^®^ prescription during the week before their orthopaedic visit were not considered to have had a CSI. Each participant was followed for 7 days, starting from the date when the orthopaedic consultation was made, and any hospital admissions were recorded. Data were obtained on hospital admissions (dates and discharge diagnoses) from the central CHS database, which includes both hospital diagnoses and diagnoses recorded in the family physician medical record. ACS was considered to have occurred when one of the following diagnoses appeared in the database as a new diagnosis from the admission period: myocardial infarction, ACS, intermediate coronary syndrome, atherosclerosis of coronary artery, coronary artery bypass graft, coronary catheterisation and angioplasty, and unstable angina pectoris.

### Statistical analysis

The baseline sociodemographic and clinical characteristics of the study participants were ascertained, using proportions for categorical variables and means and standard deviations for normally distributed continuous variables. Logistic regression was then used to compare the crude and age- and sex-adjusted odds ratio of having an ACS in the week following an orthopaedic visit, between patient visits during which an injection was or was not administered. Because ACS events were rare, the odds ratios approximate risk ratios. Individuals could be included in the analysis more than once, creating correlated observations. Therefore, robust standard errors were used, clustered by patient ID, to calculate appropriate confidence intervals and *P*-values. Because data sparsity precluded multivariable analysis, potential confounding was addressed through restriction.^30^ The analysis was repeated while stratifying on cardiovascular risk factors (that is, patients ≥75 years old, smokers, those with pre-existing ischaemic heart disease, hyperlipidaemia, hypertension, obesity, or diabetes, and those with any two or any three of these risk factors). Finally, several sensitivity analyses were conducted. To explore selection bias, the analysis was repeated including only patients who had been admitted to hospital (for any cause) within a week of their orthopaedic visit. The analysis was also repeated using various lengths of hospital stay to define an admission (that is, excluding ‘short’ 1- or 2-day admissions that may have been for elective, pre-planned procedures).

Stata (version 15.1 IC) was used to perform all statistical analyses. All data were anonymised.

## RESULTS

Between April 2012 and December 2015, 22 131 patients aged ≥50 years consulted with an orthopaedic specialist (60 856 unique visits). The mean age at the time of the visit was 70.9 years (standard deviation [SD] = 10.8), and 39.1% of the visits were by people aged ≥75 years. Males made 33.6% of visits, and 78.0% had ≥2 cardiovascular risk factors at the time of the visit (that is, obesity, smoking, hyperlipidaemia, hypertension, congestive heart failure, ischaemic heart disease, and diabetes mellitus). CSI was administered in 5.1% of all visits (3075), with 1779 patients having at least one injection over the study period. Patients who had received a CSI were more likely to be older than those who had not (mean age = 74.5 years; SD = 10.3 versus 70.7; 10.8), as well as more likely to have cardiovascular risk factors. Baseline demographic information and cardiovascular risk factors of the cohort participants are presented in Table 1.

**Table 1.** Patients’ characteristics at the time of orthopaedic visit by injection administration status

	**No CSI, N = 57 781**	**CSI, N = 3075**
**Mean age, years (SD)**	70.7 (10.8)	74.5 (10.3)
**N, 50–64 (%)**	19 409 (33.6)	634 (20.6)
**N, 65–74 (%)**	16 225 (28.1)	802 (26.1)
**N, ≥75 (%)**	22 147 (38.3)	1639 (53.3)
**N, Male (%)**	19 415 (33.6)	951 (30.9)
**N, Obesity^a^ (%)**	12 860 (22.3)	829 (27.0)
**N, Smoking^a^ (%)**	21 559 (37.3)	1157 (37.6)
**N, Hyperlipidaemia^a^ (%)**	43 989 (76.1)	2452 (79.7)
**N, HTN^a^ (%)**	27 684 (47.9)	1760 (57.2)
**N, CHF^a^ (%)**	1541 (2.7)	81 (2.6)
**N, IHD^a^ (%)**	11 415 (19.8)	747 (24.3)
**N, DM^a^ (%)**	13 365 (23.1)	752 (24.5)
**N, Mean risk factors^b^ (SD)**	2.7 (1.5)	3.0 (1.4)
**N, Mean visits per person (SD)**	3.1 (3.4)	5.0 (4.1)
**N, First visit *n* (%)**	21 443 (37.1)	504 (16.4)
**N, Second visit *n* (%)**	12 360 (21.4)	509 (16.6)
**N, Third+ visit (%)**	23 978 (41.5)	2062 (67.1)

a Based on inclusion in the CHS chronic disease register (see Supplementary Box S1 for details).

b Cardiovascular risk factors: obesity, smoking, hyperlipidaemia, hypertension, congestive heart failure, ischaemic heart disease, and diabetes mellitus. CHF = congestive heart failure. CHS = Clalit Health Services. CSI = corticosteroid injection. DM = diabetes mellitus. HTN = hypertension. IHD = ischaemic heart disease. SD = standard deviation.

Of all the orthopaedic visits, 5.4 per1000 (*n* = 329) were followed by a hospital admission (all-cause) within 7 days; 7.8/1000 (*n* = 24) of CSI visits versus 5.3/1000 (*n* = 305) of non-CSI visits, *P* = 0.06.

Of all visits, 0.4/1000 (*n* = 25) were followed by an ACS-related hospital admission. CSI visits were more likely to be followed by an acute coronary event, compared with non- CSI visits (2.27/1000 visits versus 0.31/1000, odds ratio [OR] = 7.32; 95% confidence intervals [CI] = 2.81 to 19.11; *P*<0.0001). These results remained broadly similar when adjusted for age and sex (OR = 6.62; CI = 2.52 to 17.36; *P* = 0.0001), as well as when the analysis was restricted to those with cardiovascular risk factors (Table 2).

**Table 2.** Acute coronary Syndrome within 7 days of an orthopaedic visit with a corticosteroid injection

	**Exposure**	**N, No ACS**	**N*,* ACS**	**Crude OR (95% CI)^a^**	**Adjusted OR (95% CI)^a^**
**All**	No CSI	57 763	18	-ref-	-ref-
**All**	CSI	3068	7	7.32 (2.81 to 19.11)	6.62 (2.52 to 17.36)
**Aged<65 years**	No CSI	19 407	2	-ref- (*P*= 0.03)	-ref- (*P*= 0.03)
**Aged<65 years**	CSI	633	1	15.33 (1.39 to 168.75)	15.49 (1.40 to 171.65)
**Aged ≥65 years**	No CSI	38 356	16	-ref-	-ref-
**Aged ≥65 years**	CSI	2435	6	5.91 (2.07 to 16.83)	5.95 (2.10 to 16.81)
**Female**	No CSI	38 359	7	-ref-	-ref-
**Female**	CSI	2121	3	7.75 (2.00 to 30.00)	6.42 (1.72 to 23.89)
**Male**	No CSI	19 404	11	-ref-	-ref-
**Male**	CSI	947	4	7.45 (1.95 to 28.44)	6.67 (1.72 to 25.86)
**No IHD**	No CSI	46 363	3	-ref- (*P*= 0.10)	-ref- (*P*= 0.07)
**No IHD**	CSI	2327	1	6.64 (0.69 to 63.87)	6.7 (0.86 to 52.39)
**IHD^b^**	No CSI	11 400	15	-ref-	-ref-
**IHD^b^**	CSI	741	6	6.15 (2.13 to 17.74)	6.27 (2.15 to 18.29)
**No smoking**	No CSI	36 213	9	-ref-	-ref-
**No smoking**	CSI	1913	5	10.52 (3.08 to 35.89)	8.42 (2.49 to 28.44)
**Smoking^b^**	No CSI	21 550	9	-ref- (*P*= 0.07)	-ref- (*P*= 0.08)
**Smoking^b^**	CSI	1155	2	4.15 (0.90 to 19.16)	4.15 (0.85 to 20.41)
**No hyperlipidaemia**	No CSI	13 790	2	-ref- (*P*= 0.05)	-ref-
**No hyperlipidaemia**	CSI	622	1	11.09 (1.01 to 121.85)	9.03 (0.9 to 90.15)
**Hyperlipidaemia^b^**	No CSI	43 973	16	-ref-	-ref-
**Hyperlipidaemia^b^**	CSI	2446	6	6.74 (2.37 to 19.21)	6.22 (2.18 to 17.75)
**No HTN**	No CSI	30 092	5	n/a	n/a
**No HTN**	CSI	1315	0	n/a	n/a
**HTN^b^**	No CSI	27 671	13	-ref-	-ref-
**HTN^b^**	CSI	1753	7	8.5 (3.12 to 23.15)	8.46 (3.08 to 23.21)
**No obesity**	No CSI	44 910	11	-ref-	-ref-
**No obesity**	CSI	2241	5	9.11 (2.76 to 30.07)	7.72 (2.36 to 25.24)
**Obesity^b^**	No CSI	12 853	7	-ref- (*P*= 0.06)	-ref- (*P*= 0.07)
**Obesity^b^**	CSI	827	2	4.44 (0.92 to 21.43)	4.53 (0.90 to 22.7)
**No DM**	No CSI	44 405	11	-ref- (*P*= 0.01)	-ref- (*P*= 0.02)
**No DM**	CSI	2320	3	5.22 (1.46 to 18.72)	4.72 (1.28 to 17.32)
**DM^b^**	No CSI	13 358	7	-ref-	-ref-
**DM^b^**	CSI	748	4	10.20 (2.48 to 41.94)	9.55 (2.32 to 39.24)
**No CHF**	No CSI	56 224	16	-ref-	-ref-
**No CHF**	CSI	2987	7	8.24 (3.11 to 21.79)	7.52 (2.84 to 19.90)
**CHF^b^**	No CSI	1539	1539	n/a	n/a
**CHF^b^**	CSI	81	0	n/a	n/a
**<2 risk factors**	No CSI	12 912	1	n/a	n/a
**<2 risk factors**	CSI	455	0	n/a	n/a
**Any two risk factors^c^**	No CSI	44 851	17	-ref-	-ref-
**Any two risk factors^c^**	CSI	2613	7	7.07 (2.69 to 18.57)	6.92 (2.63 to 18.26)
**<3 risk factors**	No CSI	25 910	1	n/a	n/a
**<3 risk factors**	CSI	1083	0	n/a	n/a
**Any three risk factors^c^**	No CSI	31 853	17	-ref-	-ref-
**Any three risk factors^c^**	CSI	1985	7	6.61 (2.51 to 17.37)	6.76 (2.57 to 17.83)

a *All* P*-values are* <*0.001 unless specifically stated otherwise.*

b Analysis was restricted to patients who were diagnosed with the condition at/before the index orthopaedic visit, as recorded by the Clalit Health Services chronic disease register.

c Analysis was restricted to patients diagnosed with any two or three of the above risk factors. ACS = acute coronary syndrome. CHF = congestive heart failure. CSI = corticosteroid injection. DM = diabetes mellitus. HTN = hypertension. IHD = ischaemic heart disease. OR = odds ratio.

A series of sensitivity analyses were conducted to assess potential bias. The results remained robust when short hospital admissions (that is, <1 and <2 days long) were excluded, which may have been associated with elective procedures. Finally, the results did not change substantially when the analysis was restricted to orthopaedic visits that were followed by a hospital admission (see Supplementary Table S1 for details).

## DISCUSSION

### Summary

Receiving a CSI was associated with a seven-fold increase in the risk of a subsequent ACS in an analysis of 60 856 primary care orthopaedic consultations. The results remained robust when cardiovascular risk factors were taken into consideration.

### Strengths and limitations

Over 3000 orthopaedic consultations were included in which CSI was administered; to the best of the authors’ knowledge, this is the largest report to date. Routinely collected data from a centralised medical file were utilised, assuring complete electronic capture of events. Finally, some particular features of the Israeli health system (that is, universal healthcare coverage and accessible primary care orthopaedic consultations) increase the validity of the findings; the community- and population-based sample is less prone to the selection bias compared with outpatient-based samples.

This study also has several limitations. Although the procedure of injecting a patient appears clearly in the data records, data were not available as to the content, dosage, and medical condition for which the injection was given. The injections have been presumed to be corticosteroids by excluding patients who purchased hyaluronic acid injections prior to their orthopaedic visit; however, it was not possible to differentiate between different corticosteroid products and dosages. It has also been assumed — as the injections were given by orthopaedic specialists — that they were for musculoskeletal conditions affecting soft tissues or joints. It is possible that patients suffering from inflammatory arthropathies, which themselves increase the incidence of ischaemic events,^31^ formed part of the patient population. In Israel, however, it is likely that these patients would be treated with CSIs by rheumatologists, rather than by orthopaedic specialists.

Hospital discharge data may have included elective coronary procedures, causing an increase in the number of recorded ischaemic events. However, it was possible to confirm diagnoses by extracting data from two complementary sources using both hospital and primary care records. To assess potential bias introduced by capturing elective cardiac catheterisations as new ischaemic events, the analysis was repeated excluding outcome events associated with 1- and 2-day admissions. The robustness of the association in the sensitivity analysis suggests little bias owing to misclassification of outcome events.

Finally, the relatively small absolute number of outcomes precluded the study from including all potential confounders in a single multivariable analysis. However, it was still possible to consider the possible effect of cardiovascular risk factors through both considering single risk factors and restricting the analysis to higher-risk subgroups: those patients with two and three pre-existing cardiovascular risk factors at the time of their orthopaedic visit. Indeed, physicians may have chosen CSI as a non-operative treatment for those with pre-existing cardiovascular disease or risk factors, as Table 1 suggests. The observed seven-fold increase in risk remained robust after accounting for cardiovascular risk factors, suggesting that CSI was an independent risk factor for an ACS.

### Comparison with existing literature

To the best of the authors’ knowledge, this is the first report of an association specifically between CSI and an ACS, although two large population-based studies of oral corticosteroids^14^ and all exogenous corticosteroids^15^ did show small, significant increases in ischaemic heart disease incidence. Previous reports of outcomes of CSI have generally been small and of low quality, and systemic reviews included fewer patients in total than were injected in this research.^6^^–^^8^^,^^18^^,^^19^ It is, therefore, possible that the size of this study enabled findings that have not been demonstrated elsewhere.

The pathophysiological mechanisms by which corticosteroids affect the cardiovascular system have been considered in existing literature. Their potential to exacerbate hypertension, hyperlipidaemia, hyperglycaemia, and coagulopathy are known.^11^^,^^12^^,^^32^ In addition, the presence of glucocorticoid receptors in cardiovascular tissues raises the possibility of a localised effect on atherosclerosis.^33^ Increases in blood glucose and blood pressure occur primarily in the hours and days following injections.^17^ A reduction in blood salicylate levels in the hours following CSI has also been reported,^34^ which may have been significant for the ACS patients in this study, the majority of whom had pre-existing ischaemic heart disease and are likely to have been taking aspirin. The existence of these physiological changes in the hours and days immediately after CSI led to the choice of 1 week following injection as the time period for this study.

### Implications for research and practice

This study suggests an association between receiving an intra-articular or soft-tissue corticosteroid injection and a subsequent acute coronary event. The large number of CSIs reviewed combined with accurate follow-up has detected an association between a common treatment and a rare and life-threatening side effect, not previously reported. Further research is needed to confirm the association between CSI and ACS, and to identify the patients who are at particular risk. In addition, research should investigate whether all sites of injection are equally likely to trigger ischaemia and whether other orthopaedic injectable products pose similar dangers. In the meantime, it is perhaps necessary to reconsider corticosteroids as a ‘safe’ option, particularly for patients at higher-risk.
